# Awareness of Relative Energy Deficiency in Sport Among Russian Coaches Working with Athletes of Various Levels in Athletics and Short-Track Speed Skating

**DOI:** 10.3390/sports14080341

**Published:** 2026-08-07

**Authors:** Eduard Bezuglov, Beatrisa Volel, Elizaveta Kapralova, Mikhail Vinogradov, Emina Malagych, Anna Belyakova, Anna Berdnikova

**Affiliations:** 1 Department of Sports Medicine and Medical Rehabilitation, Sechenov First Moscow State Medical University of the Ministry of Health of the Russia Federation, 119991 Moscow, Russia; e.n.bezuglov@gmail.com (E.B.);; 2 Department of Psychiatry and Psychosomatics, Sechenov First Moscow State Medical University of the Ministry of Health of the Russian Federation, 119991 Moscow, Russia; volel_b_a@staff.sechenov.ru; 3 Federal State Budgetary Scientific Institution Mental Health Research Center, 115522 Moscow, Russia; 4 Science and Research Department, Professional Football Club CSKA Moscow, 125252 Moscow, Russia; vinogradov.coach@gmail.com (M.V.); emina189@yandex.ru (E.M.); 5 Department of Neurology, Institute of Professional Education, Sechenov First Moscow State Medical University of the Ministry of Health of the Russia Federation, 119991 Moscow, Russia; asimcin@mail.ru

**Keywords:** RED-S, short-track speed skating, athletics, track and field

## Abstract

Relative Energy Deficiency in Sport (RED-S) can adversely affect athletes’ health and performance. Coaches in individual sports may help recognize warning signs and facilitate referrals, yet their awareness remains understudied, especially among high-performance professionals. A cross-sectional survey using a translated, pilot-tested, and expert-reviewed adaptation of the Lodge et al. questionnaire was conducted among 101 Russian athletics (*n* = 75) and short-track speed skating (n = 26) coaches. The primary outcome was self-reported prior awareness of RED-S. Among coaches reporting awareness, secondary outcomes were self-rated confidence and recognition of 12 adverse RED-S consequences. Of 99 coaches who answered the awareness question, 39 (39.4%; 95% CI, 30.3–49.2%) reported prior awareness. Among these 39 coaches, 19 (48.7%) rated their confidence as moderate to very high, and the mean consequence-recognition score was 5.64 ± 4.04 out of 12; 11 (28.2%) recognized at least 9 of 12 consequences. In unadjusted and multivariable analyses, no statistically significant associations were found between prior awareness and sport, sex, athletes’ competitive level, or coaching qualification, although confidence intervals were wide. These findings suggest limited prior awareness of RED-S in the surveyed sample and limited consequence recognition among coaches reporting prior awareness. They may indicate an educational gap with potential implications for athlete health. Targeted coach education should be evaluated in future studies.

## 1. Introduction

A key issue in professional sports is the long-term maintenance of athletic success. Among the most significant factors that can negatively impact athletes’ career trajectories are musculoskeletal injuries, mental health disorders, and excessive, poorly tolerated physical loads. Both the isolated and combined impact of these factors can lead to the development of a range of pathological conditions that are often difficult to diagnose but have a detrimental effect on athletic performance. Among these conditions, Relative Energy Deficiency in Sport (RED-S) and Overtraining Syndrome can be highlighted, as they are frequently part of a single continuum [1]. The term “Relative Energy Deficiency in Sport” was first introduced in 2014 by an International Olympic Committee expert group [2]. This concept replaced the previous notion of the Female Athlete Triad, which included menstrual dysfunction, eating disorders, and decreased bone mineral density [3]. This change reflected evidence that the health effects associated with low energy availability extend beyond the components of the original triad. These effects occur in both women and men [4].

In sports with predominantly aerobic demands, such as middle- and long-distance running and short-track speed skating, the issue of RED-S is particularly acute due to high energy expenditure. Data from elite endurance runners indicate that the prevalence of low energy availability may reach 30–40%, and symptoms of RED-S are directly correlated with the incidence of stress fractures and upper respiratory tract illnesses [1]. Coach awareness may therefore be important for recognizing warning signs and facilitating appropriate referral.

The cause of this syndrome is an energy deficit resulting from an imbalance between dietary energy intake and the energy required for health, daily activities, growth, and sports participation. Its consequences affect not only physical health but also psychological well-being [5]. Research on RED-S has expanded substantially since the original IOC consensus statement was published in 2014. The statement was updated in 2018 and 2023 as new evidence emerged [2,5,6], and debate continues within the scientific community [7,8].

RED-S is a significant problem for athletes of all levels due to its negative impact on physical performance, injury rates, and career longevity [9,10]. Consequently, the timely diagnosis and correction of this condition in at-risk groups is an important practical task. However, a key role in the timely identification and management of RED-S is played by the awareness of the issue not only among athletes themselves but also among those who work directly with them—including not only sports physicians but also coaches. In this context, it is important that athletes and coaches understand the symptoms, causes, and consequences of RED-S. Coach familiarity with RED-S may be particularly relevant in individual sports, where coaches often maintain sustained contact with athletes.

Despite the recognized importance of the coach’s role in early identification of RED-S, major reviews show that coaches represent a negligible proportion of participants in scientific studies on this topic—only 2.7% of all respondents [11]. Moreover, most surveys have been conducted among coaches of youth and collegiate teams, while data on specialists working with national- and international-level athletes remain scarce. For example, Logue et al. included only nine elite coaches [12], and Hamnlund et al. surveyed twelve Swedish national team coaches [13], documenting a limited ability to correctly identify the components of RED-S even within this group. No similar studies have previously been conducted in the Russian Federation.

Previous studies involving coaches and athletes have yielded conflicting results, and the number of such studies, as well as the representation of different sports within them, is very limited. Furthermore, these studies have rarely included elite-level athletes and their coaches, for whom the timely diagnosis and correction of RED-S is especially important, given the high stakes of their competitive success [14,15,16,17]. Athletics and short-track speed skating were selected because recruitment was conducted through their national federation meetings and because both are predominantly individual sports in which coaches typically maintain sustained contact with athletes. Therefore, the aim of this study was to assess self-reported prior awareness of RED-S among coaches of various levels working with athletes in two sports.

We hypothesized that self-reported prior awareness of RED-S would be low among Russian athletics and short-track speed skating coaches. Associations with sport, sex, coaching qualification, and the competitive level of the athletes coached were examined exploratorily because evidence supporting directional subgroup hypotheses was limited.

## 2. Materials and Methods

### 2.1. Participants and Recruitment

A cross-sectional study was conducted among 101 coaches working in athletics (n = 75, including 33 women) or short-track speed skating (n = 26, including 14 women) in the Russian Federation. Coaches were recruited during the annual general meetings of the All-Russian Athletics Federation and the Russian Skating Union, which are regularly attended by the majority of active coaches from these disciplines. No individual invitations were sent in advance. All 112 eligible coaches present at the two meetings were invited to participate voluntarily. Of these, 101 agreed to participate (participation rate, 90.2%), while 11 declined; reasons for declining were not recorded. Data were collected from 3 to 12 February 2025.

Inclusion criteria were: (a) currently working as a coach in athletics or short-track speed skating; (b) training athletes at national or international level; (c) providing informed consent to participate. No exclusion criteria were applied; data from all consenting coaches were included in the analysis.

Participation was fully anonymous. The questionnaire did not request any personal identifying information, and the research team had no access to the link between participants and their assigned identifiers. To prevent duplicate responses, the online survey platform automatically generated a unique identifier for each completed questionnaire, and each participant could submit the form only once. The questionnaire was administered via Yandex Forms.

The sample was obtained through convenience sampling and comprised all eligible coaches who were present at the meetings and agreed to participate (n = 101). Item-level missing responses were retained as missing; therefore, available-case denominators varied across analyses.

Exploratory subgroup analyses examined whether prior awareness differed by sport, sex, coaching qualification, and the competitive level of the athletes coached.

The subdivision based on the achievement level of the trained athletes was performed according to the athletes’ participation and/or winning places in competitions at different levels and included two subgroups: international and national.

The international level was defined as the athletes’ participation in the Olympic Games, World or European Championships, or World Cup stages. The national level was defined as the athletes’ participation in Russian Championships among adults, juniors, youth, and young athletes.

The subdivision based on the coaches’ qualification level was performed according to the system of coach qualification categories adopted in the Russian Federation. This system includes categorical division based on the competition level of the trained athletes, the coaches’ participation in educational events, authorship of methodological manuals, and possession of sporting honours. A high level (Group 1) included coaches of the highest qualification category and Honoured Coaches of the Russian Federation. A lower level (Group 2) included coaches holding the first or second qualification category and coaches without a qualification category [18].

### 2.2. Questionnaire

Awareness of RED-S was assessed using an online questionnaire based on a survey previously developed by Lodge et al. [19]. The original English-language questionnaire was translated into Russian by two independent translators and subsequently back-translated to resolve discrepancies. The adapted version was pilot-tested with ten coaches who were not part of the main sample, after which minor editorial adjustments were made. Three experts in sports medicine and exercise physiology reviewed the questionnaire for relevance to coaches’ professional activities, clarity of terminology, and applicability to the Russian sporting context. No formal quantitative content-validity index, internal-consistency analysis, test–retest reliability assessment, or construct-validation analysis was performed. In accordance with contemporary guidance on measurement validity [20], the instrument is therefore described as translated and adapted rather than validated. The complete Russian and English versions are provided as Supplementary Materials S1 and S2. The questionnaire collected demographic and professional characteristics, self-reported prior awareness of RED-S, self-rated confidence, and responses concerning warning signs, causes, and consequences of RED-S. Participants who reported no prior awareness did not complete the subsequent RED-S-specific questions. 

The primary outcome was self-reported prior awareness of RED-S, defined as a “yes” response to the awareness question. Among coaches reporting prior awareness, secondary outcomes were self-rated confidence and a consequence-recognition score. For the secondary binary confidence comparison, very high, high, and moderate responses were grouped as moderate-to-very-high confidence, whereas low and very low responses were grouped as low-to-very-low confidence (Table 1). The consequence-recognition score was the number of 12 predefined adverse consequences selected in Question 21 (range, 0–12). A score of 9/12 was the lowest integer score meeting the ≥70% criterion; because this cut-off has not been formally validated, the continuous score was also reported and the threshold analysis was interpreted as exploratory. The score was calculated for all 39 coaches reporting prior awareness; participants who selected no adverse consequences received a score of 0.

### 2.3. Statistical Analysis

An a priori sample-size calculation was not performed because recruitment was constrained to eligible coaches attending the federation meetings. The study was considered exploratory, and emphasis was placed on effect estimates and 95% confidence intervals rather than post hoc power. Missing data were not imputed; available-case analyses were used, with the denominator reported for each analysis.

Statistical analysis was performed using Python 3.11.9 (Python Software Foundation, Wilmington, DE, USA) with the pandas, numpy, scipy.stats, and statsmodels libraries. Categorical characteristics were described using absolute and relative frequencies with 95% confidence intervals for proportions calculated using the Wilson method.

For numerical variables (age, coaching experience), the following were calculated: sample size (n), mean (M), standard deviation (SD), minimum and maximum values, median, 25th and 75th percentiles, interquartile range (IQR), and a 95% confidence interval for the mean (via standard error and the t-distribution quantile with n–1 degree of freedom).

Comparison of proportions between groups was performed using 2 × 2 contingency tables. The chi-squared test with Yates’ correction was applied when all expected frequencies were ≥5; Fisher’s exact test was used when any expected frequency was <5. Two-sided *p*-values were calculated with α = 0.05. For each family of four exploratory subgroup comparisons (sport, sex, athlete level, and qualification), Holm-adjusted *p*-values were examined as a sensitivity analysis; this adjustment did not change the interpretation of any comparison. Continuous variables were compared using Welch’s independent-samples *t*-test when both group distributions were compatible with normality according to the Shapiro–Wilk test; otherwise, the Mann–Whitney U test was used.

For binary comparisons of the primary outcome, odds ratios (ORs) with 95% confidence intervals were reported. Proportions were accompanied by Wilson 95% confidence intervals.

To assess independent associations with self-reported prior awareness, multivariable logistic regression included sport, sex, athletes’ competitive level, and coaching qualification. Complete cases were used. Results are presented as adjusted odds ratios with 95% confidence intervals. Overall model fit was assessed using the likelihood-ratio test and the Hosmer–Lemeshow test. With 36 awareness events across four predictors, the model was considered exploratory and the adjusted estimates were interpreted cautiously.

## 3. Results

A total of 101 coaches participated in the survey: 54 (53.5%) men and 47 (46.5%) women; 75 (74.3%) worked in athletics and 26 (25.7%) in short-track speed skating. Age was available for 98 participants, coaching experience for 99, athlete competitive level for 93, coaching qualification for 100, and the primary awareness response for 99. The age, coaching experience, achievement level of trained athletes, and qualifications of the study participants are presented in Table 2, Table 3 and Table 4. Eight participants had missing responses to the item used for national-versus-international subgroup classification and were retained in analyses not involving athlete level.

Comparisons of age and coaching experience according to athletes’ competitive level, overall and stratified by sport, are presented in Table 5.

### 3.1. Awareness of Relative Energy Deficiency in Sport Among Participants

Awareness of RED-S was determined by the response to the question, “Have you heard of Relative Energy Deficiency in Sport (RED-S)?” Of the 99 coaches who answered this question, 39 reported prior awareness (39.4%; 95% CI, 30.3–49.2%). Prior awareness did not differ significantly by sport (short-track vs. athletics: OR = 1.18, 95% CI 0.47–2.93; *p* = 0.904), sex (female vs. male: OR = 1.75, 95% CI 0.78–3.95; *p* = 0.252), athletes’ competitive level (international vs. national: OR = 1.19, 95% CI 0.44–3.21; *p* = 0.922), or coaching qualification (Group 1 vs. Group 2: OR = 1.32, 95% CI 0.55–3.18; *p* = 0.688) (Table 6, Table 7, Table 8 and Table 9).

The multivariable logistic-regression model included 91 complete cases (36 coaches reporting prior awareness). No predictor was statistically significant (Table 10). The overall likelihood-ratio test was χ^2^(4) = 1.54 (*p* = 0.819), and the Hosmer–Lemeshow test was χ^2^(6) = 1.95 (*p* = 0.924).

### 3.2. Self-Rated Confidence and Consequence Recognition Among Coaches Reporting Prior Awareness

Among the 39 coaches reporting prior awareness, 19 (48.7%; 95% CI, 33.9–63.8%) rated their confidence as moderate to very high and 20 (51.3%) rated it as low or very low. Moderate-to-very-high self-rated confidence did not differ significantly by sport (athletics 53.6% vs. short-track speed skating 36.4%; *p* = 0.541), sex (men 44.4% vs. women 52.4%; *p* = 0.863), athletes’ competitive level (international 55.6% vs. national 40.7%; *p* = 0.470), or coaching qualification (Group 1 44.4% vs. Group 2 54.5%; *p* = 0.836).

Among these 39 coaches, the mean consequence-recognition score was 5.64 ± 4.04 out of 12 (median 6; IQR 1.5–9.0). Eleven coaches (28.2%; 95% CI, 16.5–43.8%) recognized at least 9 of the 12 adverse consequences. The most frequently recognized consequences were impaired recovery and reduced training effectiveness (61.5% each), followed by irritability and insomnia (59.0% each); infertility was recognized by 17.9%. The score was calculated across all 39 coaches reporting prior awareness, including those who selected no adverse consequences.

For both families of four exploratory subgroup comparisons, all Holm-adjusted *p*-values were 1.000 (Supplementary Table S1).

## 4. Discussion

This study surveyed experienced coaches working in athletics and short-track speed skating, with a median age of 41.5 years and median coaching experience of 16 years. Of the 99 coaches who answered the primary awareness question, 39 (39.4%) reported prior awareness of RED-S. No statistically significant differences in prior awareness were observed according to sport, sex, athletes’ competitive level, or coaching qualification.

The observed 39.4% awareness rate was lower than the 53.5% reported among Japanese track and field coaches by Tsukahara et al. (2023) [21]. Cross-study comparisons should be interpreted cautiously because differences in sampling, questionnaire content, sport context, and educational exposure may influence estimates [22]. Limited awareness or knowledge of RED-S or the Female Athlete Triad has also been reported among collegiate athletic trainers [15] and coaches in Singapore [23].

Prior awareness was not statistically significantly associated with the competitive level of the athletes coached or with sex. These findings are relevant to athletics and short-track speed skating, although athletics encompasses event groups with diverse physiological demands. Mukherjee et al. reported that 85% of 106 Singaporean coaches had never heard of or were unsure whether they had heard of the Female Athlete Triad, and none of the female coaches reporting awareness correctly identified all three components [23]. Together, these findings suggest that awareness varies across settings, but the present study cannot determine the reasons for this variation.

It is important to note that most studies investigating knowledge of RED-S among coaches have been conducted among coaches of youth and student-athletes, likely due to the greater accessibility of coaches at this level for surveying [24,25]. At the same time, studies involving coaches of international-level athletes have been insufficient to date, and their participant numbers are small—12 Swedish national team coaches in the study by Hamnlund et al. [13], 9 elite coaches in the study by Logue et al. [12]. In the present study, 21 coaches trained athletes at the international level and 72 trained athletes at the national level; athlete level was unclassifiable for eight participants because of missing responses. Thus, the sample included coaches working across national and international high-performance contexts, although direct cross-study comparisons are limited by differences in definitions and sampling methods.

Another issue in research on current sports science topics is the low involvement of coaches. In a review by Delaney et al. (246 studies published between 2018 and 2024, 38,424 participants), awareness and perception of the RED-S issue were examined in detail [11]. The authors note that only 2.7% of all participants in the included studies belonged to the coaching staff. Low involvement of coaches in scientific research was also noted in a publication by Haugen, which assessed the prevalence of studies involving coaches: out of over 1100 original studies included in the analysis, coaches participated in less than 0.5% [26].

In this context, the present study adds data on RED-S awareness among coaches, a group that remains underrepresented in sports-science research. The findings may inform future research on coach education and athlete-health support.

No statistically significant associations were observed between prior awareness and sport, sex, athletes’ competitive level, or coaching qualification in either the unadjusted or adjusted analyses. However, these findings should not be interpreted as demonstrating equivalence between groups because the confidence intervals were wide. Coaches of international-level athletes were older and had more coaching experience than coaches of national-level athletes, but the present analyses do not establish independent associations between age or experience and RED-S awareness.

Among coaches reporting prior awareness, approximately half rated their confidence as moderate to very high, while the mean consequence-recognition score was 5.64 out of 12. Self-rated confidence did not differ significantly across the examined subgroups. These findings suggest that familiarity with the term RED-S does not necessarily correspond to comprehensive recognition of its potential consequences.

The absence of clearly higher awareness among coaches with higher qualifications or international-level athletes may indicate a potential gap in continuing professional education. Nevertheless, the cross-sectional design, limited subgroup sizes, and imprecise estimates do not permit conclusions concerning causation, educational programme effectiveness, or consequences for athlete health.

Several methodological limitations warrant consideration. First, the cross-sectional design precludes causal inferences regarding coach characteristics, RED-S awareness, and athlete health outcomes. Second, convenience sampling may have introduced selection and nonresponse bias, and the respondents may not represent all Russian athletics and short-track coaches. Third, although the questionnaire underwent translation, back-translation, pilot testing, and expert review, it did not undergo formal psychometric validation or test–retest reliability assessment. Fourth, prior awareness and confidence were self-reported, while consequence recognition was assessed only among the 39 coaches who reported prior awareness. Fifth, the study did not objectively evaluate coaches’ daily screening, communication, referral, or management practices. Sixth, the short-track subgroup (n = 26), international-level subgroup (n = 21), and number of awareness events in the multivariable model were limited; consequently, the estimates were imprecise and potentially meaningful associations cannot be excluded. Finally, the sample was restricted to athletics and short-track speed skating, limiting generalizability to other sports and coaching environments.

These findings support the development and formal evaluation of targeted RED-S education for coaches. Educational content could include the principal causes and consequences of RED-S, recognition of warning signs, appropriate communication with athletes, and clear referral pathways to qualified sports medicine and nutrition professionals. Coaches should support recognition and referral rather than undertake clinical diagnosis. Prospective studies are needed to determine whether such education improves knowledge, professional practice, or athlete-level outcomes. The International Olympic Committee has developed a RED-S Clinical Assessment Tool [5]; however, its clinical application should remain within appropriately qualified multidisciplinary teams.

## 5. Conclusions

Among Russian athletics and short-track speed skating coaches, self-reported prior awareness of RED-S was limited. Among coaches reporting prior awareness, recognition of adverse RED-S consequences was also limited. No statistically significant associations were found between prior awareness and sport, sex, athletes’ competitive level, or coaching qualification, but the estimates were imprecise. These findings support the potential value of developing and evaluating targeted RED-S education for coaches.

## Figures and Tables

**Table 1:** Operational classification of self-rated confidence.

Self-Rated Confidence Category	Binary Classification Used in Secondary Analysis
Very high	Moderate-to-very-high
High	Moderate-to-very-high
Moderate	Moderate-to-very-high
Low	Low-to-very-low
Very low	Low-to-very-low

**Table 2:** Descriptive Statistics for Age and Coaching Experience.

Indicator	n	Mean	SD	Minimum	Median	IQR	95% CI for Mean
Age, years	98	43.4	12.7	21	41.5	18	40.8–46.0
Coaching experience, years	99	18.6	12.6	0	16	20	16.0–21.1

**Table 3:** Maximum Achievement Level of Trained Athletes.

Achievement Level	N	% of Enrolled Sample
International level	21	20.8
National level	72	71.3
Missing/unclassifiable	8	7.9

**Table 4:** Coaching Qualifications of Survey Participants.

Coaching Qualification	N	% of Enrolled Sample
Group 1	67	66.3
Group 2	33	32.7
Missing/unclassifiable	1	1.0

**Table 5:** Descriptive Statistics of Participant Characteristics by Athletes’ Competitive Level, Overall and Stratified by Sport.

	N		Age			Coaching Experience		
	International	National	International	National	*p*	International	National	*p*
Athletics	15	52	55.5 ± 12.0	42.5 ± 10.4	0.001	28.8 ± 13.8	17.8 ± 10.8	0.004
Short-track speed skating	6	20	42.8 ± 11.5	39.4 ± 12.5	0.545	19.4 ± 12.9	14.8 ± 9.8	0.446
Total	21	72	51.5 ± 13.0	41.7 ± 11.0	0.006	26.1 ± 13.9	17.0 ± 10.5	0.006

Note: Values are mean ± SD. Athlete-level group counts are shown in the N columns; variable-specific analyses used available age and coaching-experience data. Welch’s independent-samples *t*-test was used for overall age, short-track age, and short-track coaching experience; the Mann–Whitney U test was used for athletics age and for overall and athletics coaching experience.

**Table 6:** Self-reported prior awareness by sport.

Sport	Awareness n/N, % (95% CI)	OR (95% CI)	*p*
Athletics	28/73, 38.4% (28.1–49.8)	1.00 (reference)	0.904
Short-track speed skating	11/26, 42.3% (25.5–61.1)	1.18 (0.47–2.93)	0.904

**Table 7:** Self-reported prior awareness by coach sex.

Sex	Awareness n/N, % (95% CI)	OR (95% CI)	*p*
Male	18/54, 33.3% (22.2–46.6)	1.00 (reference)	0.252
Female	21/45, 46.7% (32.9–60.9)	1.75 (0.78–3.95)	0.252

**Table 8:** Self-reported prior awareness by athletes’ competitive level.

Athlete Level	Awareness n/N, % (95% CI)	OR (95% CI)	*p*
National level	27/70, 38.6% (28.0–50.3)	1.00 (reference)	0.922
International level	9/21, 42.9% (24.5–63.5)	1.19 (0.44–3.21)	0.922

**Table 9:** Self-reported prior awareness by coaching qualification level.

Qualification level	Awareness n/N, % (95% CI)	OR (95% CI)	*p*
Group 2	11/32, 34.4% (20.4–51.7)	1.00 (reference)	0.688
Group 1	27/66, 40.9% (29.9–53.0)	1.32 (0.55–3.18)	0.688

Notes for Table 6, Table 7, Table 8 and Table 9: Percentages were calculated using available-case denominators. Odds ratios are presented relative to the category identified as the reference. Two-sided *p*-values were obtained using chi-square tests with Yates’ correction. Holm-adjusted *p*-values across the four comparisons were 1.000 for all comparisons. OR, odds ratio; CI, confidence interval.

**Table 10:** Multivariable logistic regression for self-reported prior awareness of RED-S.

Predictor	Adjusted OR	95% CI	*p*
Short-track vs. athletics	0.97	0.37–2.55	0.954
Female vs. male	1.68	0.71–3.94	0.238
International vs. national athlete level	1.18	0.43–3.22	0.748
Qualification Group 1 vs. Group 2	1.00	0.38–2.61	0.992

Note. Adjusted odds ratios were estimated simultaneously for all four predictors (analytic N = 91; 36 awareness events). Model fit: likelihood-ratio χ^2^(4) = 1.54, *p* = 0.819; Hosmer–Lemeshow χ^2^(6) = 1.95, *p* = 0.924. OR, odds ratio; CI, confidence interval.

## Data Availability

The data that support the findings of this study are available from the corresponding author upon reasonable request.

## Supplementary Materials

The following supporting information can be downloaded at: https://www.mdpi.com/article/10.3390/sports14080341/s1, Supplementary Material S1. The full adapted questionnaire in Russian; Supplementary Material S2. The full adapted questionnaire in English; Supplementary Table S1. Raw and Holm-adjusted *p*-values for the four families of exploratory subgroup comparisons.
