# Supplementary material for: Awareness of Relative Energy Deficiency in Sport Among Russian Coaches Working with Athletes of Various Levels in Athletics and Short-Track Speed Skating
Source: Sports (Basel). 2026 Aug 7;14(8):341. doi: 10.3390/sports14080341 (PMC13517876; doi:10.3390/sports14080341)
Supplement: Supplementary file 1 [file sports-14-00341-s001.zip › sports-4408983-supplementary.pdf]

Supplementary Material S1. The full adapted questionnaire in Russian

Раздел 1. Общая информация

1. Ваш возраст (в годах): _____

2. Ваш пол:
   - женский
   - мужской

3. Какой вид спорта вы представляете в настоящее время: _____

4. Стаж работы тренером (в годах): _____

5. На каком уровне вы занимались спортом:
   - не занимался этим видом спорта
   - массовый разряд (1 взрослый и ниже)
   - кандидат в мастера спорта
   - мастер спорта России
   - мастер спорта международного класса
   - заслуженный мастер спорта

6. Самый высокий уровень спортивных достижений ваших спортсменов:
   - призер Олимпийских Игр и чемпионата мира
   - участник Олимпийских Игр и чемпионата мира
   - призер чемпионата Европы или этапа Кубка мира
   - участник чемпионата Европы и этапа Кубка мира
   - призер чемпионата России среди взрослых
   - участник чемпионата России среди взрослых
   - призер чемпионата России среди юниоров, юношей и молодежи
   - участник чемпионата России среди юниоров, юношей и молодежи

7. Какая тренерская квалификация у вас на момент опроса:
   - тренер без категории
   - тренер второй категории
   - тренер первой категории
   - тренер высшей категории
   - заслуженный тренер России

8. Какое количество тренировок обычно бывает в недельном цикле у ваших спортсменов самого высокого уровня?
   - до 10
   - 11-15
   - более 15

9. Сколько часов в неделю обычно тренируются при подготовке к соревнованиям ваши лучшие спортсмены?
   - до 10 часов
   - 10-15 часов
   - 16-20 часов
   - более 20 часов

10. Бывают ли у ваших спортсменов в недельном тренировочном цикле дни полностью свободные от тренировок?
    - нет
    - очень редко (один раз в месяц и реже)
    - иногда (не чаще 1-3 раз в месяц)
    - один раз в неделю -два раза в неделю
    - от 1 до 3 дней в зависимости от тренировочного цикла

Раздел 2. Информированность о синдроме относительного дефицита энергии в спорте

11. Слышали ли вы о синдроме относительного дефицита энергии в спорте (RED-S)?
    - да
    - нет

    При ответе «нет» опрос заканчивается, при ответе «да» опрос продолжается

12. Откуда вы узнали о синдроме относительного дефицита энергии (RED-S)?
    - от других тренеров
    - посещая семинары и прочие образовательные мероприятия
    - из социальных сетей
    - из научных статей и исследований
    - от медицинского персонала, который работает (работал) с моими спортсменами
    - от медицинских работников, которые не являются членами моей команды
    - другое: _____

13. Как вы оцениваете свою осведомленность о синдроме относительного дефицита энергии (RED-S):
    - очень высокая
    - высокая
    - средняя
    - низкая
    - очень низкая

Раздел 3. «Красные флаги» о синдроме относительного дефицита энергии (RED-S)

14. Знаете ли вы о "красных флагах"(тревожных симптомах), связанных с синдромом относительного дефицита энергии (RED-S):
    - да
    - нет

15. Если да, то отметьте, какие из следующих признаков вы считаете "красными флагами" синдрома относительного дефицита энергии (RED-S) (можно выбрать несколько вариантов):
    - стрессовые повреждения костной ткани
    - нарушения сна
    - потеря веса в течение последних недель
    - снижение или отсутствие аппетита
    - снижение мышечной силы
    - повторяющиеся инфекционные заболевания
    - олигоменорея (нарушение цикла с промежутками между месячными 40 дней и более), аменорея (отсутствие менструаций в течение 6 месяцев при нормальном цикле ранее)
    - снижение скорости восстановления
    - другое: _____

16. Основной причиной синдрома относительного дефицита энергии в спорте является:
- нарушение питания
- аменорея (отсутствие менструаций в течение 6 месяцев, при нормальном цикле ранее)
- низкое потребление энергии (недостаточное потребление на фоне высокого уровня расхода энергии за счет тренировок)
- анемия (снижение уровня железа в организме)
- низкая минеральная плотность костной ткани

17. Как вы оцениваете важность своевременного распознавания этих признаков:
- очень важно
- важно
- не очень важно
- совсем не важно

Раздел 4. Восприятие и последствия синдрома относительного дефицита энергии (RED-S)

18. Считаете ли вы, что недостаточное потребление энергии (недоедание) может негативно сказаться на спортивных результатах:
- да
- нет
- не знаю

19. Как вы думаете, внимание тренера к «идеальному» весу спортсмена помогает ему понять как он/она может показать лучшие результаты в своем виде спорта:
- да
- нет
- не знаю

20. Знаете ли вы о физических и психологических последствиях синдрома относительного дефицита энергии (RED-S):
- да
- нет

21. Если да, пожалуйста, перечислите некоторые из них:
- снижение выносливости
- улучшение выносливости
- депрессия
- улучшение настроения
- бесплодие
- увеличение полового влечения
- снижение полового влечения
- повышенный риск получения травм
- раздражительность
- улучшение спортивных результатов
- бессонница
- увеличение мышечной силы
- снижение мышечной силы
- ухудшение координации
- улучшение координации
- снижение концентрации внимания
- повышение концентрации внимания
- снижение скорости восстановления

- ускорение скорости восстановления
- сниженная результативность тренировок
- повышенная результативность тренировок
- свой вариант: _____

Раздел 5. Поддержка и обучение

22. Насколько важно, по вашему мнению, проводить обучающие семинары о синдроме относительного дефицита энергии (RED-S) для спортсменов и тренеров:
- очень важно
- важно
- не очень важно
- совсем не важно

23. О каких темах, связанных с синдромом относительного дефицита энергии (RED-S) вы хотели бы получить информацию:
- о его влиянии на травматизм
- о его влиянии на спортивные результаты
- о его влиянии на репродуктивную функцию
- о его влиянии на ментальное здоровье
- о профилактике этого состояния
- о основных принципах диагностики этого состояния
- о основных признаках коррекции этого состояния

Supplementary Material S2. The full adapted questionnaire in English

Chapter 1. Participant demographics

1. Age (in years): _____

2. Sex:
   - female
   - male

3. Athletic discipline: _____

4. Total years of coach experience: _____

5. Level of participation:
   - no experience as athlete in this athletic discipline
   - first senior degree or lesser
   - sub-master athlete
   - master of sports of Russia
   - master of sports of Russia of International Class
   - merited master of sports

6. Highest level of sporting achievements of our athletes:
   - Olympic medalist and/or World championship medalist
   - Olympian competitor and/or World championship competitor
   - European championship medalist and/or World Cup series medalist
   - European championship competitor and/or World Cup series competitor
   - Russian senior championship medalist
   - Russian senior championship competitor
   - Russian junior championship medalist
   - Russian junior championship competitor

7. Current coaching qualification:
   - non-categorized coach
   - level 2 coach
   - level 1 coach
   - high-performance coach
   - Merited Coach of Russia

8. How many training sessions are usually in a weekly cycle for your highest-level athletes:
   - < 10
   - 11-15
   - > 15

9. Weekly training hours for highest-level athletes during pre-competition period:
   - < 10 hours
   - 10-15 hours
   - 16-20 hours
   - > 20 hours

10. Do your athletes have days in their weekly training cycle that are completely free from training:
    - no

- very rarely (once a month or less)
- sometimes (no more than 1–3 times a month)
- once a week
- twice a week
- 1 to 3 days depending on the training cycle

Chapter 2. Knowledge about Relative Energy Deficiency in Sport

11. Have you ever heard about "Relative Energy Deficiency in Sport (RED-S)":
- yes
- no

If you answer "No", the survey ends. If you answer "Yes", please proceed to the next question.

12. How did you learn about Relative Energy Deficiency in Sports (RED-S):
- other coaches
- educational program
- social media
- research articles
- medical staff who work/worked with my athletes
- medical staff who are not part of my team
- other: _____

13. How do you assess your level of awareness regarding Relative Energy Deficiency in Sports (RED-S):
- very high
- high
- moderate
- low
- very low

Chapter 3. Relative Energy Deficiency in Sports (RED-S) "red flags"

14. Do you know the "red flags" (warning symptoms) of Relative Energy Deficiency in Sports (RED-S):
- yes
- no

15. If yes, please check which of the following signs you consider to be "red flags" of Relative Energy Deficiency in Sports (RED-S) (multiple choices allowed):
- bone stress injuries
- sleep disturbances
- weight loss over the recent weeks
- decreased or lack of appetite
- decreased muscle strength
- recurrent infectious diseases
- oligomenorrhea (menstrual cycle disturbances with intervals of 40 days or more), amenorrhea (absence of menstruation for 6 months in a previously normal cycle)
- impaired recovery
- other: _____

16. The main cause of Relative Energy Deficiency in Sports (RED-S) is:
- eating disorder(s)
- amenorrhea (absence of menstruation for 6 months in a previously normal cycle)
- low energy availability (insufficient energy intake relative to a high level of energy expenditure from

training)
- anemia (low iron level)
- low bone mineral density

17. How would you rate the importance of early detection of these signs:
- very important
- important
- not very important
- unimportant

Chapter 4. Perception and consequences of Relative Energy Deficiency in Sports (RED-S)

18. Low energy availability (under-eating) can negatively affect athletic performance:
- yes
- no
- I don't know

19. A coach's attention to an athlete's "ideal" weight helps the athlete understand how to achieve better results in their sport:
- yes
- no
- I don't know

20. Are you aware of the physical and psychological consequences of Relative Energy Deficiency in Sports (RED-S):
- yes
- no

21. If yes, please list some of them (check all that apply):
- decreased endurance
- improved endurance
- depression
- improved mood
- infertility
- increased libido
- decreased libido
- increased risk of injuries
- irritability
- improved athletic performance
- insomnia
- increased muscle strength
- decreased muscle strength
- impaired coordination
- improved coordination
- decreased concentration
- increased concentration
- decreased recovery
- increased recovery
- decreased training efficiency
- increased training efficiency
- other: _____

Chapter 5. Support and education

22. How important to conduct educational workshops on Relative Energy Deficiency in Sports (RED-S) for athletes and coaches:
- very important
- important
- not very important
- unimportant

23. Which topics related to Relative Energy Deficiency in Sports (RED-S) would you like to receive information about:
- its impact on injury rates
- its impact on athletic performance
- its impact on reproductive function
- its impact on mental health
- prevention of this condition
- key principles of detection of this condition
- key approaches to managing and correcting this condition

Supplementary Table S1. Raw and Holm-adjusted p-values for the four families of exploratory subgroup comparisons.

Family / comparison	Group 1 (n/N, %)	Group 2, reference (n/N, %)	Test	Raw p	Holm-adjusted p
Family 1. Self-reported prior awareness of RED-S (n = 99)					
Sport	Short-track, 11/26 (42.3%)	Athletics, 28/73 (38.4%)	χ^2 (Yates)	0.904	1.000
Sex	Female, 21/45 (46.7%)	Male, 18/54 (33.3%)	χ^2 (Yates)	0.252	1.000
Athlete level	International, 9/21 (42.9%)	National, 27/70 (38.6%)	χ^2 (Yates)	0.922	1.000
Qualification	Group 1, 27/66 (40.9%)	Group 2, 11/32 (34.4%)	χ^2 (Yates)	0.688	1.000
Family 2. Moderate-to-very-high self-rated confidence (n = 39)					
Sport	Short-track, 4/11 (36.4%)	Athletics, 15/28 (53.6%)	χ^2 (Yates)	0.541	1.000
Sex	Female, 11/21 (52.4%)	Male, 8/18 (44.4%)	χ^2 (Yates)	0.863	1.000
Athlete level	International, 5/9 (55.6%)	National, 11/27 (40.7%)	Fisher exact	0.470	1.000
Qualification	Group 1, 12/27 (44.4%)	Group 2, 6/11 (54.5%)	χ^2 (Yates)	0.836	1.000
Family 3. Consequence-recognition score, 0–12 (n = 39)					
Sport	Short-track, 5.55 ± 4.70	Athletics, 5.68 ± 3.84	Mann–Whitney U	0.987	1.000
Sex	Female, 5.71 ± 4.10	Male, 5.56 ± 4.08	Mann–Whitney U	0.955	1.000
Athlete level	International, 6.56 ± 4.64	National, 5.37 ± 4.09	Mann–Whitney U	0.438	1.000
Qualification	Group 1, 5.85 ± 3.97	Group 2, 5.09 ± 4.53	Mann–Whitney U	0.603	1.000
Family 4. Recognition of ≥ 9 of 12 consequences (n = 39)					
Sport	Short-track, 4/11 (36.4%)	Athletics, 7/28 (25.0%)	Fisher exact	0.694	1.000

Sex	Female, 6/21 (28.6%)	Male, 5/18 (27.8%)	χ² (Yates)	1.000	1.000
Athlete level	International, 4/9 (44.4%)	National, 7/27 (25.9%)	Fisher exact	0.409	1.000
Qualification	Group 1, 7/27 (25.9%)	Group 2, 4/11 (36.4%)	Fisher exact	0.696	1.000

Note. Each family comprised four comparisons (sport, sex, athletes' competitive level, and coaching qualification); the Holm–Bonferroni step-down procedure was applied within each family. Proportions are shown as events/available responses (%); consequence-recognition scores are shown as mean ± SD. Chi-square tests used Yates' continuity correction; Fisher's exact test was used when any expected frequency was below 5. Within every family the smallest raw p-value (0.252, 0.470, 0.438, and 0.409, respectively) corresponded to a Holm-adjusted p-value of 1.000, and no comparison reached statistical significance at $\alpha = 0.05$. Denominators vary because item-level missing responses were retained as missing.
